# The effect of environmental change, planned and unplanned life events on the long-term outcome of common mental disorders

**DOI:** 10.1007/s00127-023-02520-1

**Published:** 2023-07-10

**Authors:** Peter Tyrer, Conor Duggan, Min Yang, Helen Tyrer

**Affiliations:** 1 School of Public Health, Sichuan University, Chengdu, China; 2 Faculty of Health, Art and Design, Swinburne University of Technology, Melbourne, Australia; 3 Division of Psychiatry, Imperial College, London, UK; 4 Department of Forensic Psychotherapy, University of Nottingham, Nottingham, UK

**Keywords:** Environmental interventions, Common mental disorders, Personality disorder, Nidotherapy, Social prescribing

## Abstract

**Purpose:**

To examine the nature of positive and negative environmental change on clinical outcome in 210 patients presenting with anxiety and depression and followed up over 30 years.

**Methods:**

In addition to clinical assessments, major environmental changes, particularly after 12 and 30 years, were recorded in all patients by a combination of self-report and taped interviews. Environmental changes were separated into two major groups, positive or negative, determined by patient opinion.

**Results:**

In all analyses positive changes were found to be associated with better outcome at 12 years with respect to accommodation (*P* = 0.009), relationships (*P* = 007), and substance misuse (*P* = 0.003), with fewer psychiatric admissions (*P* = 0.011) and fewer social work contacts at 30 years (*P* = 0.043). Using a consolidated outcome measure positive changes were more likely than negative ones to be associated with a good outcome at 12 and 30 years (39% v 3.6% and 30.2% v 9.1%, respectively). Those with personality disorder at baseline had fewer positive changes (*P* = 0.018) than others at 12 years and fewer positive occupational changes at 30 years (*P* = 0.041). Service use was greatly reduced in those with positive events with 50–80% more time free of all psychotropic drug treatment (*P* < 0.001). Instrumental positive change had greater effects than imposed changes.

**Conclusions:**

Positive environmental change has a favourable impact on clinical outcome in common mental disorders. Although studied naturalistically in this study the findings suggest that if harnessed as a therapeutic intervention, as in nidotherapy and social prescribing, it would yield therapeutic dividends.

## Introduction

Although it is widely appreciated that environmental factors have a major role in the outcome of mental illness most of the evidence is retrospective. Principally, the influence of deprivation, abuse and neglect early in life has been shown to be responsible for, or at least heavily implicated in, the later manifestation of mental disorder, both in adolescence and adult life [1–4]. The study of life events has also reinforced awareness that the environment is a trigger for many illness episodes [5–9], but not necessarily a reason for greater psychiatric consultations [10].

Prospective studies of environmental change sometimes have the advantage of agency; they can be manipulated and otherwise altered to influence mental illness. The evidence that investment in occupational interventions [11, 12], ‘green care’ and other activities linked to the natural world [13, 14], and drama [15] can all have benefits for mental health is strong but still needs more vigorous evaluation.

But in evaluating the positive effects of environmental change in mental disorders there are few studies that evaluate the relative effects of environment and standard treatments, especially over the longer term. The opportunity to compare the relationship between clinical interventions and environmental factors came about in a long-term follow-up study of common mental illness, the Nottingham Study of Neurotic Disorder.

In this study, carried out between 1983 and 2019, 210 patients presenting at general practice psychiatric clinics with the most common mental disorders, anxiety and depression, were first involved in a 10 week randomised trial of treatments [16] and then followed up over a 30 year period. The most detailed data were obtained at the 12 year and 30 year follow-up points in the study.

## Method

The Nottingham Study of Neurotic Disorder recruited patients from general practice psychiatric clinics in Nottingham between 1983 and 1987. These clinics were set up primarily to improve liaison with general practitioners and were commonly used in the latter years of the twentieth century to extend community care [17, 18] and reduce psychiatric admissions [19]. As patients were seen at an early stage they could be regarded as more representative of mental illness in the community than those seen in standard out-patient clinics.

In the first phase of the study 210 patients were randomised to drug treatment (*n* = 84) (separated into the antidepressant, dothiepin [*n* = 28], the anti-anxiety drug, diazepam [*n* = 28], and placebo [*n* = 28]), cognitive behaviour therapy (*n* = 84), and self-help (*n* = 42). Each of these were given for 6 weeks and then tapered so that at 10 weeks-the patients were on no therapy [16]. Normal clinical care was then followed but if patients had improved on their randomised treatment they were given the option of continuing in the same mode (i.e. drugs, psychological treatment or self-help) subsequently and most did so in the first 2 years after randomisation, with the one exception being the prescription of benzodiazepines, which were prescribed similarly in all groups [20]. After 2 years, follow-up care continued as necessary and the study became a cohort one.

The inclusion criteria for the original trial were (i) no active psychiatric treatment at entry, (ii) informed consent, (iii) a diagnosis of either GAD, panic disorder or dysthymic disorder (or any mixture of these), using the Structured Clinical Interview for DSM-III [21] and (iv) no history of other presumed independent psychiatric illness (schizophrenia, bipolar disorder or alcohol or drug addiction).

### Follow-up

Patients were followed up and had clinical assessments 16, 32, 52 and 104 weeks after randomisation and at 5 years had their service contacts recorded by examination of notes but without being interviewed [22]. Full clinical and environmental assessments were carried out at 12 and 30 years.

The primary outcome at 30 years was the absence of a DSM diagnosis of any affective (including anxiety) disorder [23]. As long-term outcome includes many elements beyond clinical symptoms a combined symptomatic, social, and service outcome was generated in advance of any analysis to allow good, intermediate, and poor outcomes to be generated.

The 30-year follow-up also allowed a better assessment of long-term outcomes. One of the difficulties in comparing the impact of environmental changes, particularly in the context of standard therapeutic interventions, is that of time. Standard drug and psychological treatments tend to have their main effects within a matter of weeks or days, whereas the effects of environmental changes, unless they are specifically formulated as short term (e.g. as in randomised trials of interventions such as singing [24] are usually delayed. As in the Nottingham Study of Neurotic Disorder major data were collected at 12 and 30 years the option of testing together both clinical and environmental outcomes was taken up (Table 1).

**Table 1:** Time points of clinical and environmental outcomes over the 30-year study

Outcomes	Baseline	1 year	2 years	12 years	30 years
Clinical					
Self-rated anxiety (HADS-A)	x	X	X	x	x
Observer-rated anxiety (BAS)	x	X	X	x	x
Self-rated depression (HADS-D)	x	X	X	x	x
Observer-rated Depression (MADRS)	x	X	X	x	x
General Neurotic Syndrome Rating Scale (GNSS)	x				
Personality status severity (PAS)	x		X	x	x
Environment and global					
Preferred treatment				x	x
Life events scale	x		X		
Major life changes	x		X	x	x
Positive impact of changes				x	x
Negative impact of changes				x	x
Social function (SFQ)				x	x
Life chart				x	x
Global outcome				x	x
NDOS				x	x

### Outcomes recorded over the 30 year follow-up

There were four key points at which a range of clinical and environmental outcomes were assessed over the time course of the study (Table 1).

Many of the outcome measures were closely related and so their specific data are not recorded here. These include the MADRS scale for depression [25], the Brief Anxiety Scale [26], both subdivisions of the Comprehensive Psychopathological Rating Scale (CPRS) [27], and the Hospital Anxiety and Depression Scale [28]. The general neurotic syndrome, a Galenic syndrome [29] recorded in the study [23], also correlates highly with other measures, particularly personality status. For this reason, only one symptomatic outcome, the scores on the CPRS, was included in a putative combined outcome scale. Other broader items included were social function and scores on the Neurotic Disorder Outcome Scale (NDOS) [30], a 10 point measure of longitudinal outcome.

Before analysis the subjects were separated into good, intermediate and poor outcome groups. The criteria for each element of the scale are indicated in Table 2.

**Table 2:** Association between effects of environment change and overall clinical outcome

Change effect	Overall outcome			*P* value	
	Good *n* (%)	Intermediate *n* (%)	Poor *n* (%)	Unadjusted^a^	Adjusted^b^
0–12 years					
				** < 0.0001**	**0.007**
Positive change	18 (39.1)	27 (58.7)	1 (2.2)		
Negative change	1 (3.6)	16 (57.1)	11 (39.3)		
No change	30 (29.4)	51 (50.0)	21 (20.6)		
Total	49	94	33		
13–30 years					
				0.409	0.302
Positive change	16 (30.2)	25 (47.2)	12 (22.6)		
Negative change	1 (9.1)	6 (54.5)	4 (36.4)		
No change	1 (9.1)	7 (63.6)	3 (27.3)		
Total	18	38	19		

Bold indicates all levels of significance beyond *P* < 0.05

NB. The overall clinical outcome was defined by the following criteria determined from previous study data:

Good outcome: SFQ < 6 & CPRS < 10 & NDOS ≤ 2 [31–33]

Poor outcome: SFQ ≥ 10 & CPRS ≥ 16 & NDOS ≥ 4 [31, 33, 34]

Intermediate: All those not in the above two categories

To qualify for a good or poor outcome all three scores had to be in range shown above

^a^Fisher’s exact test

^b^Adjusted for personality disorder status using multinomial models with intermediate outcome as reference group

The environmental hypothesis at the start of the study in 1983 was a general one, that ‘environmental factors would have at least an equivalent influence on long-term outcome than clinical ones’. A subsidiary hypothesis was that personality disorder would be more susceptible to negative environmental factors than clinical symptoms, as this had been found in previous studies [35, 36].

#### Personality status

Personality assessment was made at baseline by previously trained independent researchers (all psychiatrists) using the Personality Assessment Schedule (PAS) [37–39]. The basic principle behind the PAS is that personality disturbance is best viewed as a spectrum between normal personality function and severe personality dysfunction and this has now been adopted as the new ICD-11 classification of personality disorder [40]. A previous study examined the baseline PAS scores and the criteria for the ICD-11 classification were adjusted to be concordant with ICD-11 [41].

### Environmental outcomes and service costs

Assessments of environmental changes recorded over the 30 year study period are summarised in Table 1.

#### Preferred treatment

After the assessments made at 12 and 30 years each patient was asked to rate which treatment in the past 12- or 18-year-period, respectively had helped them most. The options given were (a) drug treatment, (b) psychological treatment (of any sort), (c) self-help (including self-help groups, books or other media, (d) no treatment was superior to any other, and (e) nothing helped. Patients were also asked follow-up questions to amplify their responses and the answers noted down. These helped to decide on the impact of each environmental change.

#### Major life changes

A life chart was constructed for each patient at 12 and 30 years and all relevant events noted together with their date of onset. A previous study covering the first 2 years of the Nottingham study had shown that those with some personality disorders had more, mainly negative, life events than others [36]. The event choices were made from the Paykel Life Events scale [42].

#### Perceived positive events

Perceived positive events were recorded from a combination of answers to questions at interview, the information from the life charts and events scales and the comments about preferred treatment. In almost all cases these responses were congruent and there was no need for another independent assessment.

#### Perceived negative events

This followed the same principle as for positive events and also used the same three sources of data.

As a separate component to the study the environmental changes were classified as either (i) spontaneous or unavoidable (e.g., death of close relative, serious illness, standard date of retirement), (ii) created by decisions made by the patient, (iii) created by individuals on behalf of the patient, and (iv) created by others independently of the patient, were separated into positive instrumental changes, those carried out for the intended benefit of the patient, or positive imposed changes, those instigated by others with no patient involvement. A similar distinction was made for negative changes.

#### Differentiation of environmental changes and relationship to life events

The environmental changes chosen in the methodology were a combination of both independent and contextual life events and decision making that led to important change that could not easily be defined as a life event (e.g., a personal decision to end alcohol abuse). For this reason the assessments made went beyond the methodology of life event literature, which generally has been focused on threats [43] rather than positive change. But if one uses the broadest definition of a life event as any life change that has an impact on psychological well-being [44] then all changes are life events. A small proportion of the changes also came about as a consequence of therapeutic intervention by a mental health professional and so were not typical life events. Planned environmental change to improve mental health is formalised as nidotherapy [45, 46] and examination of the data by all the authors confirmed that some of the interventions could be classed as nidotherapy. In examining the elements of change, an attempted consensus was made after examining all the data, including short voice recordings of the interviews after they had been completed.

#### Social function

Social function was measured using the Social Functioning Questionnaire (SFQ) [32], an 8 item scale with scores between 0 and 3 for each item, with a maximum score of 24. High scores indicate poor social function.

The primary clinical outcome, a simple one recommended at the beginning of the study by Sir Richard Doll [23] was the absence or presence of a significant DSM-III diagnosis (excluding adjustment and simple stress reactions and simple phobias). The principal secondary outcome was the score on the Social Functioning Questionnaire (SFQ), a measure which is correlated strongly with personality status [33, 47].

At 12 years all service costs were recorded using a combination of questions at interview and examination of the general practice notes (approved in advance as part of the ethical submission). At 30 years notes were not examined so exact details of out-patient appointments could not be determined, but admissions to hospital, suicide episodes, social work and day centre contacts, and details of drug treatments were obtained at interview [48].

### Statistical analysis

The analysis was carried out for the periods of 0–12 years and 13–30 years separately, to examine the impact of environmental changes and nidotherapy during the first 12 years and the remaining 18 years on the combined outcome scales, personality status and use of services at the end of the two periods respectively. Due to missing data on different outcomes, the first 12-year period had available data points in the analysis between 175 and 182, and between 75 and 89 in the second 18-year period. Previous analysis of the cohort data suggested many of the missing data could be regarded as occurring at random [23]. Considering only small number of missing data at each period, list-wise deletion approach was used for all analyses in this study.

For continuous outcomes, geometric means and standard deviations by environment change level (no change, positive change and negative change) were presented. One-way analysis of variance was performed to test differences in the geometric means among change levels due to heterogeneity in variances among change levels, with 95% confidence intervals by change levels presented. For discrete outcomes, percentage by environment change level was presented and differences between change levels tested using Fisher’s exact test. Since descriptive analysis showed no significant difference in patients’ age and gender proportion by environment change levels, no adjustment for age and sex was made for comparison of all outcomes. However, in examining the impact of environmental changes on the combined outcome scale, we adjusted for personality status using multinomial models.

We used IBM SPSS v19 for most of the analysis, with MLwiN v3.03 for multinomial models.

## Results

These are separated into five sections.

### Environment and overall outcome

At 12 years, 176 subjects provided data, with 49 (27.8%) having a good outcome and 33 (18.8%) a poor one. Those with positive environmental changes were significantly more likely to have a good outcome (39.1%) and less likely a poor outcome (2.2%), compared with 3.6% good outcome and 39.3% poor outcome in those who had negative environmental change (*P* < 0.007 after adjustment) (Table 2). At 30 years, following attrition through death and failure to follow-up, only 85 subjects had data and although the results were similar with 16 (30.2%) having a good outcome after positive instrumental change compared with 1 (9.1%) after negative change this was not a significant difference (adjusted *P* = 0.3) (Table 2).

### Type of environmental change and outcome

A much larger proportion of the cohort had positive environmental changes that could not be clearly identified as instrumental or imposed. In the first 12 years positive changes of accommodation (*P* = 0.009), relationships (*P* = 0.007), and substance use (*P* = 0.003) were all significantly associated with more good outcomes and less poor outcomes than no change group; at 30 years these differences did not reach significance (Table 3).

**Table 3:** Impact of types of positive and negative environment change on overall clinical outcome

Type of changes	Positive change	Negative change	No change	*P*^a^ value (adjusted)^b^
	*n* (%)	*n* (%)	*n* (%)	
0–12 years				
Accommodation changed	*N* = 27	*N* = 7	*N* = 102	**0.009**
Good outcome	12 (44.4)	0 (0)	30 (29.4)	
Intermed outcome	15 (55.6)	6 (85.7)	51 (50.0)	
Poor outcome	0 (0)	1 (14.3)	21 (20.6)	
Relationship changed	*N* = 24	*N* = 16	*N* = 102	**0.007**
Good outcome	11 (45.8)	1 (6.3)	30 (29.4)	
Intermed outcome	13 (54.2)	10 (62.5)	51 (50.0)	
Poor outcome	0 (0)	5 (31.3)	21 (80.8)	
Substance use behaviour changed	*N* = 4	*N* = 11	*N* = 102	**0.003**
Good outcome	1 (25.0)	0 (0)	30 (29.4)	
Intermed outcome	2 (50.0)	3 (27.3)	51 (50.0)	
Poor outcome	1 (25.0)	8 (72.7)	21 (20.6)	
Occupation changed	*N* = 16	*N* = 1	*N* = 100	0.058
Good outcome	6 (37.5)	0 (0)	29 (29.0)	
Intermed outcome	10 (62.5)	0 (0)	51 (51.0)	
Poor outcome	0 (0)	1 (100.0)	20 (20.0)	
13–30 years				
Accommodation changed	*N* = 13	*N* = 4	*N* = 11	0.598
Good outcome	4 (30.8)	0 (0)	1 (9.1)	
Intermed outcome	6 (46.2)	2 (50.0)	7 (63.6)	
Poor outcome	3 (23.1)	2 (50.0)	3 (27.3)	
Relationship changed	*N* = 33	*N* = 5	*N* = 10	0.449
Good outcome	10 (30.3)	0 (0)	1 (9.1)	
Intermed outcome	15 (45.5)	4 (80.0)	7 (63.6)	
Poor outcome	8 (24.2)	1 (20.0)	2 (27.3)	
Substance use behaviour changed	*N* = 5	*N* = 3	*N* = 11	0.170
Good outcome	1 (20.0)	0 (0)	1 (9.1)	
Intermed outcome	3 (60.0)	0 (0)	7 (63.6)	
Poor outcome	1 (20.0)	3 (100.0)	3 (27.3)	
Occupation changed	*N* = 31	*N* = 3	*N* = 11	0.339
Good outcome	12 (38.7)	1 (33.3)	1 (9.1)	
Intermed outcome	15 (48.4)	2 (68.7)	7 (63.6)	
Poor outcome	4 (12.9)	0 (0)	3 (27.3)	

Bold indicates all levels of significance beyond *P* < 0.05

^a^Fisher’s exact test

^b^Due to zero case in each type of the changes, multinomial models did not converge and no significant test was performed

### Impact of personality disorder

Patients diagnosed with personality disorder at 12 years had fewer positive events (26.1%) that those with no personality disorder (73.9%) (*P* = 0.018) (Table 4), mirroring the findings after 2 years [36], with occupational changes showing the largest differences between personality disorder and no disorder (*P* = 0.041) (Table 4).

**Table 4:** Relationship between different environment changes and personality status

Type of environment changes	Positive change	No change	Negative change	*P*^a^ value
	*n* (%)	*n* (%)	*n* (%)	
All changes				
0–12 years	*N* = 46	*N* = 102	*N* = 28	
No personality disorder	34 (73.9)	56 (54.9)	12 (42.9)	**0.018**
Personality disorder	12 (26.1)	46 (45.1)	16 (57.1)	
13–30 years	*N* = 53	*N* = 11	*N* = 11	
No personality disorder	27 (50.9)	3 (27.3)	6 (54.5)	0.365
Personality disorder	26 (49.1)	8 (72.7)	5 (45.5)	
Accommodation changed				
0–12 years	*N* = 27	*N* = 102	*N* = 7	
No personality disorder	20 (74.1)	56 (54.9)	3 (42.9)	0.138
Personality disorder	7 (25.9)	46 (45.1)	4 (57.1)	
13–30 years	*N* = 13	*N* = 11	*N* = 4	
No personality disorder	7 (53.8)	3 (27.3)	2 (50.0)	0.449
Personality disorder	6 (46.2)	8 (72.7)	2 (50.0)	
Relationship changed				
0–12 years	*N* = 24	*N* = 102	*N* = 16	
No personality disorder	18 (75.0)	56 (54.9)	6 (37.5)	0.057
Personality disorder	6 (25.0)	46 (45.1)	10 (62.5)	
13–30 years	*N* = 33	*N* = 11	*N* = 5	
No personality disorder	18 (54.5)	3 (27.3)	3 (60.0)	0.247
Personality disorder	15 (45.5)	8 (72.7)	2 (40.0)	
Substance use behaviour changed				
0–12 years	*N* = 4	*N* = 102	*N* = 11	
No personality disorder	2 (50.0)	56 (54.9)	5 (45.5)	0.906
Personality disorder	2 (50.0)	46 (45.1)	6 (54.5)	
13–30 years	N = 5	N = 11	N = 3	
No personality disorder	2 (40.0)	3 (27.3)	0 (0)	
Personality disorder	3 (60.0)	8 (72.7)	3 (100.0)	0.787
Occupation changed				
0–12 years	*N* = 16	*N* = 100	*N* = 1	
No personality disorder	13 (81.3)	55 (55.0)	0 (0)	**0.041**
Personality disorder	3 (18.8)	45 (45.0)	1 (100.0)	
13–30 years	*N* = 31	*N* = 11	*N* = 3	
No personality disorder	17 (54.8)	3 (27.3)	3 (100.0)	0.055
Personality disorder	14 (45.2)	8 (72.7)	0 (0)	

Bold indicates all levels of significance beyond *P* < 0.05

^a^Fisher’s exact test

### Environmental change and service costs

The data showed that psychiatric admissions (*P* = 0.011) and social work contacts (*P* = 0.013) were significantly greater in those with negative environmental changes. The most marked difference was in the number of months without any psychotropic drugs being taken, with those having positive events had nearly twice the period being drug free compared with the other two groups at 12 years (Table 5).

**Table 5:** Use of services by environmental changes (geometric mean, SD and confidence interval)

	Positive *N*: geometric mean ± SD 95% CI	Negative *N*: geometric mean ± SD 95% CI	Unchanged *N*: geometric mean ± SD 95% CI	*P* value^a^
12 years				
No. of psychiatric OP appts	46: 0.76 ± 1.06 0.43–1.17	28: 2.06 ± 2.44 0.93–3.83	107: 0.91 ± 1.89 0.56–1.33	0.057
No. of psychiatric admissions	46: 0.015 ± 0.11 0.0–0.046	29: 0.216 ± 0.44 0.065–0.39	107: 0.058 ± 0.28 0.005 – 0.84	**0.011**
Day centre (days)	46: 0.17 ± 1.13 0.0–0.46	29: 0.62 ± 1.54 0.15–1.27	107: 0.26 ± 1.17 0.09–0.46	0.217
No. of suicide attempts	46: 0.11 ± 0.43 0.0–0.23	29: 0.36 ± 0.75 0.11–0.67	107: 0.22 ± 0.62 0.11–0.34	0.184
Months free of psychotropic drugs	46: 109.06 ± 1.004 87.89–135.27	28: 58.56 ± 2.73 35.28–96.80	107: 67.86 ± 2.76 52.05–88.36	**0.000**
No. social work contacts	46: 0.19 ± 0.62 0.04–0.37	29: 1.01 ± 2.90 0.23–2.30	109: 0.29 ± 0.94 0.14–0.47	**0.013**
30 years				
Day centre (days)	53: 0.07 ± 0.41 0.0–0.17	11: 0.59 ± 3.63 0.0–2.93	11: 0.00 ± 0.00 0.0–0.0	0.058
No. of suicide attempts	53: 0.25 ± 0.94 0.04–0.49	11: 0.65 ± 1.10 0.06–1.55	11: 0.21 ± 0.38 0.00–0.46	0.887
Months drug free	53: 69.04 ± 7.30 38.59–122.89	11: 13.87 ± 13.07 2.12–70.03	11: 44.33 ± 10.92 9.48–195.0	0.094
No. social work contacts	53: 0.10 ± 0.53 0.0–0.23	11: 0.51 ± 1.52 0.0–1.60	11: 0.79 ± 1.76 0.0–2.28	**0.043**

Bold indicates all levels of significance beyond *P* < 0.05

^a^ANOVA on geometric means

### Effects of therapeutically induced environmental change

The joint assessments of all the positive instrumental environmental changes also revealed that at least 6 of the 46 interventions in the first 12 years and 22 of the 45 interventions in the second 18 years were initiated by a mental health professional or a person with mental health skills. This type of intervention is now part of the interventions described as social prescribing and nidotherapy. The interventions with these numbers were too small to analyse but they do indicate that mental health staff have a potential role in initiating positive change.

Many of these changes and their major consequences were difficult to classify easily. Examples are given below.

#### Example 1: positive instrumental change

A housing advisor with persistent anxiety symptoms treated with medication became increasingly unhappy with the bureaucracy of her job and, after discussing options with her husband, decided to retire early and move to a seaside county 250 miles away. She and her husband settled in a village and quickly integrated into the local community. At 30-year follow-up she had lost all her original symptoms, had no psychiatric contact, was a valued member of a volunteer group helping older people in the village, and both she and her husband hosted village events in supporting a local charity.

#### Example 2: negative instrumental change

A patient living in an inner city ward gradually accumulated additional members of her family when they needed accommodation and so asked her housing association to be moved off so she could live in a flat on her own. But when this was arranged she was placed in a retirement complex on the edge of Nottingham where she became completely isolated and because transport was poor in the area she was very seldom visited. When assessed at follow-up, she took a very long time to answer the door as ‘I never have a visit nowadays’. Initially she had viewed the move as a positive one but it was clearly classed as negative at 30 years, after she had been living in the flat for 5 years, and regretted bitterly that she had made the housing request.

#### Example 3: positive imposed change

A man who had worked for an engineering company for most of his working life became increasingly stressed. He had frequent depressive episodes and became fearful of many of the tasks that were demanded of him. These became more complex and involved more travelling time than previously, especially when the company was put under economic pressure and laid off staff. Eventually he was made redundant. In retrospect, he thought this was the best thing that had ever happened to him. He got his life into balance, developed very good relationships with his grandchildren and moved to a smaller house needing little maintenance. At follow-up, he described himself as fully recovered with no contact with mental health services.

#### Example 4: negative imposed change

A highly anxious secretary with no friends maintained a level of stability by support from colleagues at work and her family. The support of her colleagues became even more important when her father died. Shortly after this she was made redundant when the company reorganised. At the time of follow up she was severely agoraphobic and virtually housebound, had self-harmed twice, was drinking to excess and taking benzodiazepines and two antidepressants continuously.

These examples indicate a mixture of occupational, accommodation and relationship changes. Some of the instrumental changes even had a spontaneous element to them.

#### Example 5: a fortuitous mixture of positive and negative events

A very anxious and depressed woman with frequent attacks of panic was never able to maintain regular employment and had a series of failed relationships. She was frequently suicidal and many of her self-harm episodes led to general hospital admission because they were so severe. On one of these occasions, she was looked after by a male nurse and they developed a cordial relationship. She continued to remain emotionally unstable and 2 years later took another large overdose and was readmitted to the same general hospital. By coincidence she was looked after by the same nurse who had seen her on the previous episode. Their relationship continued to grow and they remained in contact after her discharge. Shortly afterwards he proposed and they married. Their relationship continued to prosper but eight years later her husband died. But her mental stability remained reinforced and at follow-up, she was only taking benzodiazepines for anxiety, and broke off the follow-up interview at 6 pm to drink a toast to her husband, a ritual that had been carried out regularly since he died.

These descriptions indicate that a lot more needs to be done to describe these events systematically and comprehensively and new terminology may be needed.

## Discussion

### Main findings

In a well-known pioneering study Eysenck used national insurance data (Equitable Life Society) to show that the outcome of ‘neurosis’, as defined at that time, was the same as that achieved by formal psychotherapy. In both groups, about third of the people in each group improved and the rest either remained the same or became worse [49]. The results of the Nottingham Study were very similar to those of Eysenck after 30 years despite apparent advances in treatment. Close to a half still had a significant DSM diagnosis at assessment, with those with personality pathology having worse outcomes [23]. There was also little evidence that treatments given over the course of the study had major impact on outcome [48].

In interpreting the data the impact of environmental change features strongly. Those with positive environmental events had most of their pathology halved compared with those who had negative environmental events at 12 years and although the differences were less significant at 30 years account has to be taken of the smaller numbers in the 30-year follow up (Table 3). Those with common mental disorders who recognise environmental change and life events as positive have been shown in other studies to have better outcomes [50, 51].

The results give a strong indication that positive environmental factors, including a wide admixture of life events, are at least as effective, if not greater, than specific treatment interventions in common mental disorders. The clinical status of the patients overall was no better after 30 years than at baseline and yet there was clear evidence that some environmental changes had significant effects on outcome. Although it could be claimed that all the positive changes were standard life events some were much more complex as the case examples above indicate. When patients regarded the advice about smoking and drug behaviour, usually given in the first or second assessment, as critical in their improvement at 30 years, that cannot be regarded as a simple life event. The fact that this particular memory was so important after this long period indicates the potential power of the first psychiatric interview, when a comprehensive assessment is followed by a formulation of the problem in all its aspects.

The smaller number of positive environmental changes in those with personality disorder could have contributed to the generally worse outcome in this group, a finding that is now very well-known but not fully understood [52]. These patients also incurred greater costs than others at 12 years [53]. It is reasonable to suppose that if more positive events can generated in those with a tendency to negative interactions, as in personality disorder, these consequences could be reversed, particularly if environmental changes can reinforce personality strengths [54] that are protective in the longer term.

### Strengths and limitations

Although there are many studies on the impact of the environment on health, with a growing number promoting access to nature and green spaces, these are focused mainly on general well-being rather than direct management of significant mental illness. They also focus on one type of environmental change instead of the full range available. Our study is unusual in three respects; (a) examining all types of environmental change, (b) having simultaneous records of changes in environment and clinical status, and (c) following the trajectories of patients for 30 years. There is one distant comparison. In a systematic review of health promotion in schools, the effects of interventions focused on environmental change, nutrition, exercise, safety, substance use and family life were compared. Those promoting mental health rather than trying to prevent ill-health, and those focusing on environmental change were the most effective [55]. This accords with our findings but the studies are not strictly comparable.

There are several important limitations to this study. The systematic examination of types of environmental change, particularly the separation of instrumental and imposed change, was not part of the original study and necessarily much of the data have been obtained retrospectively. It could therefore be influenced by bias, but we have tried to keep this to a minimum by allowing patients to drive the separation of positive from negative changes from their own reports and not by inference. By taking a longer time frame, interventions that might first be perceived as negative or neutral may be understood to be positive in the long-term, and vice-versa. This is the main advantage of a longer follow-up period.

#### Role of therapeutic intervention in positive instrumental change

Positive instrumental environmental change can be assisted by health practitioners in many ways. At its simplest level simple advice may be sufficient, such as explaining the advantages of giving up smoking or reducing hazardous drinking. At the other extreme someone with social disadvantage with very limited resources has very few options available and successful ones have to be worked for assiduously. Some of the interventions which led to environmental change in the Nottingham study were clearly therapist engendered. Although the benefits of these were too small to be significant they do suggest that if formal environmental interventions such as nidotherapy had been given during the course of the 30 years greater improvement might have been possible. Often the benefits of nidotherapy are delayed [56] and so it is preferable to use a long time course in their evaluation.

In this study there was no attempt to manipulate the environment deliberately to improve significant mental illness. It is becoming clear that this type of intervention could be of great value but at present has not been well studied. The two most common forms of environmental intervention are social prescribing and nidotherapy. In social prescribing patients can be referred to a link worker with knowledge of environmental options in the immediate locality. Such interventions may often be of value to the patient but research is this area has not yielded strong results because of barriers to implementation and concern over training needs [57–59] and one recent meta-analysis suggested it was both clinically ineffective and cost-ineffective [60]. Nidotherapy overlaps with social prescribing but is more complex [61], and has been shown to be of promise in several studies, including two randomised trials [56, 62–64] and also to improve social function in severe mental illness [65] but more larger scale studies are needed. The development of instrumental interventions more generally needs attention and will assist in this work. As personality function is likely to be closely linked to environmental change the measurement of personality status is also important in these enquiries [66, 67]. More attention needs to be paid to the potential cost-savings of such interventions, as they are generally cheap and when they offer equivalent outcome to more expensive alternatives they are to be fostered [63, 68].

## Conclusion

The finding that, despite sometimes continuous and varied treatments and repeated care appointments, the effects of positive environmental change were greater than standard management in terms of clinical outcome, must call for a re-evaluation of the therapeutic strategies for treating common mental illness, particularly in the presence of personality disorder [29]. Only a small proportion of the positive changes in our study were created by therapists in services, but those that did take place, including many that were spontaneous, led to even more improvement than those receiving formal psychiatric treatment. There are, therefore, good reasons to believe that if the focus of management shifted from personal intervention for symptoms to an evaluation of environmental needs and their interaction [61], and consequent attention to those needs, the aim of fuller recovery can be achieved. This will not be easy, as chronic environmental deprivation is itself resistant to improvement but in treatments such as nidotherapy there is growing evidence that change engineered by a different attitude both to care and in society can yield long-term gains [62, 69]. But such changes have to made after careful planning, and other linked approaches such as social prescribing need to have a more rigorous structure linked to systematic assessment if they are to succeed.

## Data Availability

Data supporting the study findings are available from the corresponding author and Professor Yang upon reasonable request.
